# The Axillary Fossa: An Uncovered Hidden Site as a New Alternative for Cardiac Pacemaker and Defibrillator Implantation

**DOI:** 10.19102/icrm.2019.100404

**Published:** 2019-04-15

**Authors:** Esteban M. Kloosterman, Jonathan Rosman, Murray Rosenbaum

**Affiliations:** ^1^Department of Cardiology, Lynn Heart and Vascular Institute, Boca Raton Regional Hospital, Boca Raton, FL, USA; ^2^Charles E. Schmidt College of Medicine, Florida Atlantic University, Boca Raton, FL, USA

**Keywords:** Cardiac device implant techniques, cardiovascular implantable electronic device implantation, extracellular matrix, pacemaker implantation

## Abstract

Inadequate thickness of subcutaneous tissue, pectoralis muscle wasting, and/or a lack of availability of subpectoral space can become significant issues in patients with or requiring cardiovascular implantable electronic devices (CIEDs). This is particularly concerning but not exclusive in the elderly population, who may experience discomfort and hypersensitivity of the site as well as the potential for erosion and an increased risk of infection. Thus, the use of an alternative location, the axillary fossa, offers several advantages that make it a suitable option. Specifically, it usually has a preserved fat pad (even in thin patients); is unperturbed by arm movement; is not directly exposed to contact; is easily accessed with no significant compromise of neurovascular structures; and is near the conventional subclavicular sites, with enough lead length to reach in case of the need for generator replacement. Here, we present a series of five patients, including details of their anatomy and the implant techniques used. Two underwent device replacements, with one of them presenting with significant ongoing site discomfort and the other with extreme tissue thinning, respectively. Two patients with no significant fat layer or pectoral muscle wasting had new pacemakers implanted. Lastly, a biventricular implantable cardioverter-defibrillator generator was reimplanted in a younger patient who had issues with protrusion and discomfort in the setting of thin subcutaneous tissue and the subpectoral space being occupied by a large breast implant. In conclusion, the use of the axillary fossa as a new alternative CIED implantation site, using the proposed implant technique, appears feasible and safe and demonstrated excellent results related to patient comfort and adequate device cover in five cases.

## Introduction

The lack or inadequate thickness of subcutaneous tissue can arise as a significant problem in patients with or who require cardiovascular implantable electronic devices (CIEDs). This applies particularly but not exclusively in the elderly population, as they more often than not have tissue fragility and the presence of a thin subcutaneous fat layer, which can lead to pressure from the implanted device being placed on the subcutaneous tissue. With this, the potential for erosion and infection as well as local discomfort and hypersensitivity may arise.^1^ In these situations, the subpectoral location of the pocket is a valid option. However, there are still patients who either have a thin/atrophic pectoralis muscle, which would allow for excessive device protrusion, or else they are already symptomatic while having the device placed at this site (eg, in one of the cases we present below). We thus thought that the use of an alternative location, the axillary fossa, might be a viable alternative. This site has several advantages that make it a suitable option. First, it usually has a preserved fat pad, even in thin individuals. Additionally, it remains unperturbed by arm movement and is not directly exposed to contact. Furthermore, the site is easily accessible, with no significant compromise of neurovascular structures and is in close proximity to the conventional subclavicular sites, with enough lead length to reach in the event generator replacement is required. We have previously gained experience with the lateral chest wall for device placement primarily with subcutaneous implantable cardioverter-defibrillators (S-ICDs), yet the proposed new location of the axillary fossa, although lateral, is far more superiorly located, within reach of existing leads in the case of device replacement and with additional perceived advantages, as described below.

**Figure 1:**
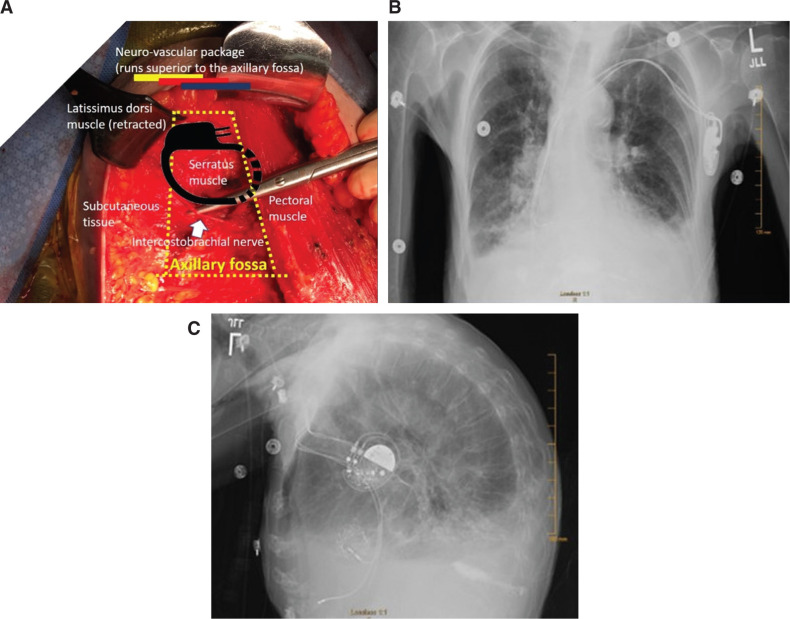
**A:** The anatomic boundaries of the axillary fossa and the proposed site of pacemaker implant. Importantly, the picture was obtained for illustration purposes from a right mastectomy case with lymph node removal that exposed the anatomic area of interest; this image should not be taken to show that this is the dissection needed for the pacemaker implant. **B and C**: Cases 3 and 4 postprocedure, respectively. Chest radiograph depicting the lateral axillary position of the generators showing the profile of the pacemaker and biventricular ICD in the posteroanterior view, respectively.

The anatomic boundaries of the axillary fossa space are somewhat triangular in shape. First, the serratus anterior muscle covers the rib cage. This is the anchoring site of the posterior aspect of the device (the back of the pocket). The lateral aspect is bordered by the teres major muscle and part of the latissimus dorsi. Medially located is the border of the pectoralis muscle. Anteriorly (covering the device’s front aspect) is the subcutaneous tissue. Superiorly, but not adjacent and not in contact with the device position, runs a major neurovascular package including the brachial plexus, axillary artery, and vein with major branches **(Figure 1A)**.

The device generator is then laterally located in the axillary fossa as opposed to anteriorly subpectorally, as shown in the chest radiograph **(Figures 1B and 1C)**.

## Methods

We herein present five cases performed using the axillary fossa for implantation. Described are two pacemaker generator changeouts, two new pacemaker implantations, and a biventricular ICD generator reimplantation. All patients were female, with four being elderly with extreme subcutaneous and muscular tissue thinning and one being middle-aged with slim subcutaneous tissue and a developed pectoralis muscle lacking available submuscular space, respectively.

The cases were performed in the electrophysiology laboratory and operating room. Conscious sedation was managed by the anesthesia service; one case (case 4) required general anesthesia.

### Case 1

An 81-year-old white female presented to the clinic with a history of sick sinus syndrome, who had undergone an initial pacemaker implant in 1989 and several replacements—with the most recent being in 2016—all performed elsewhere. The patient came to our practice with complaints of significant discomfort at the pacemaker site occurring since her last replacement. The site was also aggravated by certain movements and arm positions, adversely affecting her quality of life and ability to sleep.

**Figure 2:**
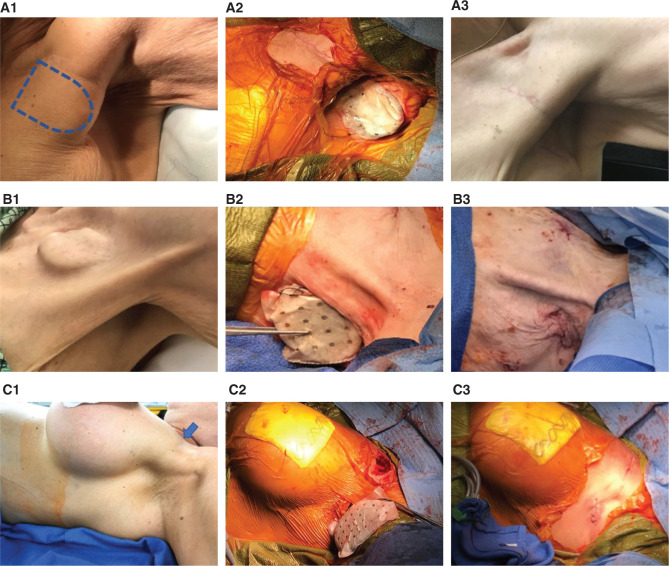
**A**: Case 1. **B**: Case 3. **C**: Case 4. Columns from left to right show baseline presentation, leads tunneled transpectorally to the axillary fossa and reconnected to the new generator after placement in an ECM bioresorbable envelope, and device inside the pocket, respectively. **A1:** Pacemaker silhouette is marked at the margin of the pectoralis muscle. **B1:** Protrusion with extreme tissue thinning and evidence of pectoralis muscle wasting. **C1:** Arrow indicating site of previous implant (subcutaneous leads in place; subpectoral breast implants). **A3:** Pacemaker site at one month after surgery. **B3 and C3**: Pacemaker site immediately after implant.

The pacemaker site appeared well-healed, with no evidence of infection or inflammation. The suture line was preserved, but the overall subcutaneous tissue was extremely thin, with evidence of subpectoral placement of the device. The pacemaker generator protrusion was readily evident and palpable at the margin of the pectoral muscle (still submuscular) **(Figure 2)**.

The most common approach for this type of pocket revision would have been to move the device medially, keeping it in the subpectoral plane and possibly anchoring it to the rib periosteum to avoid migration. The concern with that approach, however, was that the patient’s arm was already extremely sensitized (having had prior interventions as well) and an adequate comfort level could not likely have been achieved via this option. Instead, we elected to use a novel alternative site: the axillary fossa. This location is exempt from being affected by any arm movement and seems neutral to any body position or postural change. The pacemaker generator was also replaced for a smaller footprint unit (Medtronic^®^ ADDRS1; Medtronic, Minneapolis, MN, USA) **(Figure 2)**.

### Case 2

**Figure 3:**
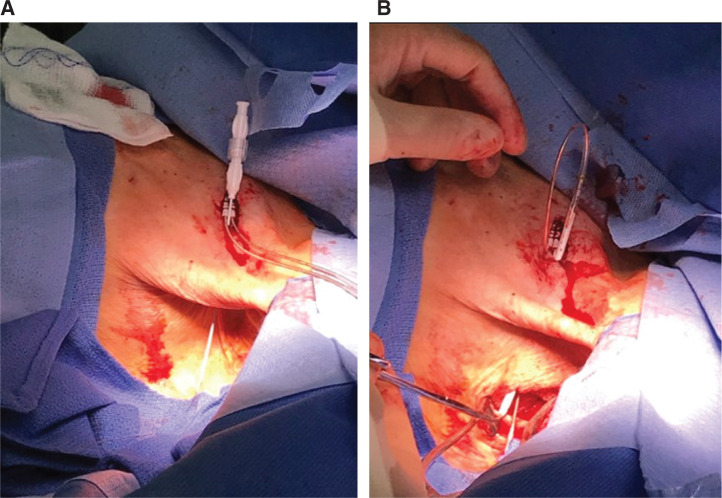
Case 2. Example of transpectoral tunneling of the leads technique. **A**: A micropuncture kit was used to access the axillary fossa space. Note that the puncture was performed near the lead insertion site, angled tangential to the chest wall and perpendicular to the pectoral muscle. **B:** A 9-Fr introducer with a proximal end cut was used to easily move the leads down into the axillary pocket.

A 96-year-old white female requiring a new pacemaker implant for sick sinus syndrome and tachy-brady syndrome with symptomatic four-second pauses presented. She had readily evident poor subcutaneous and muscular tissue. A smaller-volume generator unit was used (St. Jude Medical^®^ model 5820; Abbott Laboratories, Chicago, IL, USA). Because of her poor subcutaneous and muscular tissue, we elected to implant in the axillary fossa **(Figure 3)**.

### Case 3

A 92-year-old white female with an initial pacemaker implant occurring in 2005 for sick sinus syndrome and generator replacement in 2010 presented. She eventually developed chronic atrial fibrillation with slow ventricular response. The device was at an elective replacement indicator with extreme tissue thinning and site discomfort. The pacemaker was downgraded to a single-chamber device (St. Jude Medical^®^ model 5620; Abbott Laboratories, Chicago, IL, USA) and the atrial lead was capped. Again, due to extreme tissue thinning and site discomfort, we elected to implant in the axillary fossa **(Figure 2B)**.

### Case 4

A 49-year-old Hispanic female with a history of aortic valve disease requiring aortic valve replacement was seen. She developed progressive cardiomyopathy, left ventricular dyssynchrony, and end-stage heart failure requiring enrollment on a transplant list. A VVI ICD was implanted three months later with an ejection fraction (EF) of 25%. Given the widening of the QRS with interventricular conduction delay and New York Heart Association functional class IV symptoms, she was upgraded to a biventricular pacemaker and ICD two months later. The patient had large subpectoral breast implants near where the device was implanted, creating a subcutaneous left infraclavicular area. Of note is that she had a slim/athletic body build with thin subcutaneous tissue. Her clinical condition dramatically improved postimplant, and after more than a year of medical therapy, her EF normalized and she was able to resume an active lifestyle. Her device site, however, continued to cause significant discomfort, affecting her quality of life and physical capacity.

After several requests for the removal of the device, it was decided that it would be turned off and subsequent follow-ups regarding her EF would be completed. After six months of maintaining a normal EF on no biventricular pacing, the generator was removed two years following the initial implant, at the patient’s request. The leads remained in place. Unfortunately, she subsequently developed progressive congestive heart failure with deterioration of her EF to 30% as well as worsening mitral regurgitation. She was admitted to the hospital and, after diuresis and stabilization of her condition, she underwent biventricular ICD reimplantation in the axillary fossa at two months after removal of the previous generator (Medtronic^®^ model DTMA1QQ; Medtronic, Minneapolis, MN, USA) **(Figure 2C)**.

### Case 5

An 81-year-old white female with a history of syncope and insertable cardiac monitor (ICM) placement (Medtronic^®^ model LNQ11; Medtronic, Minneapolis, MN, USA) requiring a pacemaker implant for tachy-brady syndrome with paroxysmal atrial fibrillation and a symptomatic 4.8-second conversion pause associated with recurrent syncope presented. She had minimal subcutaneous and muscular tissue as well as readily evident ICM protrusion and discomfort. The ICM was removed and a standard volume generator unit was implanted (Medtronic^®^ model W1DR01; Medtronic, Minneapolis, MN, USA) in the axillary fossa.

## Technique

During surgery, it is important to have the patient properly positioned on the operating room table to facilitate access to the axillary fossa. We recommend that the patient be prepared with the arm of the device site in a board at a 90° angle to expose the axillary fossa. For draping, we prefer the two-piece U-shape universal sheaths, which allow for selective exposure of the area of interest with adequate covering of the rest. Lateral cradle movement of the table to further expose the axillary fossa is beneficial.

### Step 1

#### For new implants

The subclavicular percutaneous access approach, cephalic vein cutdown, or a described percutaneous transaxillary approach to the axillary vein may be used. However, this technique does not account for the axillary fossa device placement.^2–4^ Once access is obtained for the first two approaches, a 2-cm incision can be performed, taking it down to the muscularis fascia where the leads will be anchored in the usual fashion and then covering of the suture sleeves under a muscle flap should be performed, leaving the ends of the leads free.

#### For device replacement with pocket repositioning

A 2-cm to 3-cm incision over the existing scar line may be used if the patient’s anatomy allows. Then, the leads and device must be confirmed to be free from fibrous adhesions. The suture anchoring sleeves may be removed to minimize the hardware bulk and cover the area very proximal to the insertion site of the leads under a muscle flap, leaving the ends of the leads free.

### Step 2

#### Tunneling technique

Although there are several ways to tunnel the leads down to the axillary fossa space, we used the one described below as a consistent and safe method. This technique applies for both of the above-described previous approaches.

A micropuncture needle (with aspirating syringe) is used to transect, in a tangential fashion, close to the rib cage, the pectoral muscle from the anchoring site of the leads to the axillary fossa **(Figure 3)**. If any blood is aspirated in the syringe, the needle should simply be pulled back and the angle slightly changed, redirecting it away from the intersected vessel. Aiming toward the center of the axillary fossa should help to avoid any major vasculature (ie, axillary vein/artery are usually superior to the site). Once the needle is through the skin of the axillary fossa, this can act as a good guideline of the incision site, ideally 3 cm to 4 cm in length at the center of the axillary fossa perpendicular to the midaxillary line **(Figures 2 and 3)**. The incision should be taken down to the muscularis fascia of the serratus muscle and a subcutaneous pocket can be performed with essentially an inferior dissection, having a 1-cm to 2-cm area for superior dissection if needed. The pocket is irrigated with a copious amount of antibiotic solution. If, during the dissection of the pocket, the intercostobrachial nerve is encountered, it can be spared, but, if inadvertently compromised, there is little if any clinical consequence. Potential complaints of numbness of the upper medial aspect of the affected arm after surgery have been reported when accessing this area using a different approach **(Figure 1)**.^2^

A micropuncture catheter is advanced over the wire, which can then subsequently be exchanged for a 0.35 typical J-guidewire. A 9-French (Fr) sheath and introducer are advanced over the wire through the pectoral muscle. At this point, the dilator is removed (leaving the J-wire in place), allowing for the advancement of a second J-guidewire. One of the J-wires is selected and the 9-Fr dilator and sheath are readvanced through the muscle to the fossa. The wire is then removed and the top valve/portion of the sheath is cut. One of the lead’s ends can be then advanced into the sheath, snugging the distal electrode into it. Now, the end of the sheath across the muscle into the fossa can be gently pulled, allowing the lead to pass through. The sheath is then reloaded over the dilator, which is used over the remaining wire to gain access to the second lead with the same technique. With both leads positioned across the pectoral fossa, the new pacemaker device is ready to be connected **(Figure 3)**.

We chose the smaller-volume pacemaker generators available on the market (St. Jude Medical^®^ models 5620 and 5829 from Abbott Laboratories, Chicago, IL, USA and Medtronic^®^ model AADRS1 from Medtronic, Minneapolis, MN, USA), yet, in the fourth case, a biventricular ICD with a rounded contour was able to be adequately seated in the space (Medtronic^®^ model DTMA1QQ; Medtronic, Minneapolis, MN, USA), while, in the fifth case, a standard pacemaker size was used (Medtronic^®^ model W1DR01; Medtronic, Minneapolis, MN, USA).

The device and leads for all cases were placed in an envelope made of a bioresorbable extracellular matrix (ECM) that was hydrated and embedded in 1 g of vancomycin or 80 mg of gentamicin antibiotic solution for infection prevention. This material conforms to the pacemaker generator to stabilize the can and leads, after being anchored to the muscle to prevent migration. Additionally, the ECM remodels and revascularizes over time, providing another layer of tissue, which, in these cases, was thought to help with patient comfort (CanGaroo^®^; Aziyo Biologics, Inc., Roswell, GA, USA). Dermabond™ (Ethicon, Somerville, NJ, USA)/Steri-strips™ (3M, St. Paul, MN, USA) and an occlusive dressing were applied.

## Results

**Figure 4:**
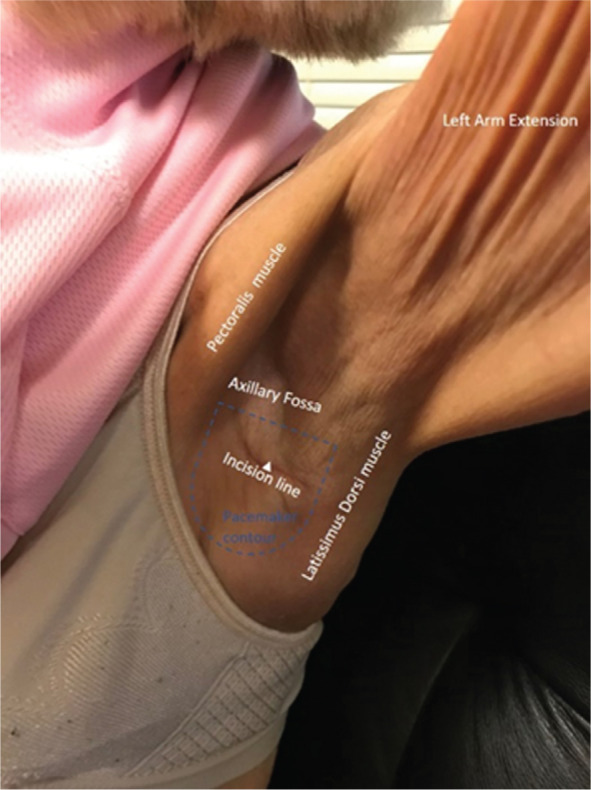
Case 5. Results seen at about six weeks after axillary fossa DDD pacemaker implantation. Observe the well-healed axillary incision line, with no protrusion of the device, with extension of the arm, pectoral, and latissimus dorsi muscles without affecting the device pocket site.

In all patients, the procedures were well-tolerated and the devices were well-seated, with adequate device function including defibrillation threshold testing in the biventricular ICD case. There were no vascular or other significant complications and no infections. After an initial period of one week to two weeks of common wound-healing-related soreness, all patients had unobstructed, full range of motion on the ipsilateral arm **(Figure 4)**. The patient in case 4 did experience left upper arm swelling thought to be due to heart failure (associated with edema of the lower extremities as well as a negative ultrasound for thrombosis) and transient numbness of the middle aspect of the arm postoperatively with minimal residual at two weeks after surgery and no pain or functional mobility impairment.

Between two weeks and three months of follow-up, there were no infections or complaints of discomfort at the site. Cases 1 and 4 experienced significant improvement of their preexisting symptoms.

## Discussion

We present an initial experience of a new cardiac device implantation site at the axillary fossa with a limited number of patients, yet the foundation of the technique and anatomy are very predictable and reproducible. Although this technique was conceived for use in elderly patients with minimal subcutaneous and/or thin muscular tissue, we showed that it could also be applied in other settings for cosmetic or comfort reasons and in cases when the subpectoral space is not available.

The axilla appears as a protected space subject to no direct contact with other body structures or external elements like undergarments, other clothing, and seat belts, which present as the most common sources of discomfort or wound stress in the typically used infraclavicular space. Different from the lateral chest wall (a site used commonly for S-ICD implantation), the axillary space is not subject to direct pressure while sleeping or lying on that site. Specifically, the axillary fossa site lacks significant movement and is unaffected by surrounding muscle groups. As a result, the leads’ motion or torqueing stress is minimal.

Possible complications to consider related to this approach other than those related to standard device implant in the subclavicular region with subcutaneous or subpectoral pockets may include injury of the intercostobrachial nerve, resulting in numbness of the upper medial/internal aspect of the related arm, which could be temporary or permanent, without affecting the motor function of the limb. Although unlikely to be compromised, there is also a major neurovascular package, including the brachial plexus and axillary artery and vein, with their branches in the superior aspect of the intended axillary fossa pocket area that should be avoided.

A best effort is always made to match the patient’s body profile to the generator can using the smallest-volume pacemaker and the softest rounded-contour biventricular ICD shape to fit in the available space. Not all devices are suitable for all patients, and assessments must be performed on an individual basis. Further experience regarding long-term patient comfort and other unforeseen interactions would be needed.

Of note, the use of intracardiac transcatheter devices (eg, Micra™ Transcatheter Pacing System from Medtronic, Minneapolis, MN, USA) could certainly overcome some of these issues, yet, at present, the device may not be optimally used in patients with preserved atrioventricular synchrony or may add an additional procedural risk in patients with existing adequately functioning leads.

The presented experience and description of this novel approach in a group of patients without many other options does not pretend to be claiming a position as a new standard in practice but rather should be taken as a subject of scrutiny by other operators who may decide to try it when warranted.

## Conclusion

Here, the axillary fossa is proposed as a new alternative pacemaker and ICD implantation site in patients with thin subcutaneous tissue, pectoralis muscle wasting, a lack of availability of the subpectoral space, and/or who are experiencing discomfort and hypersensitivity at the conventional implant site. The technique, at least in the short-term, appears to be feasible and safe and to demonstrate excellent results with regard to patient comfort and adequacy of device cover.
